# Endometriosis as a risk factor for ovarian or endometrial cancer — results of a hospital-based case–control study

**DOI:** 10.1186/s12885-015-1821-9

**Published:** 2015-10-21

**Authors:** Stefanie Burghaus, Lothar Häberle, Michael G. Schrauder, Katharina Heusinger, Falk C. Thiel, Alexander Hein, David Wachter, Johanna Strehl, Arndt Hartmann, Arif B. Ekici, Stefan P. Renner, Matthias W. Beckmann, Peter A. Fasching

**Affiliations:** 1 Department of Gynecology and Obstetrics, Erlangen University Hospital, Friedrich Alexander University of Erlangen–Nuremberg, Comprehensive Cancer Center Erlangen-EMN, Erlangen, Germany; 2 Biostatistics Unit, Department of Gynecology and Obstetrics, Erlangen University Hospital, Friedrich Alexander University of Erlangen-Nuremberg, Erlangen, Germany; 3 Current address: ALB FILS KLINKEN GmbH, Goeppingen, Germany; 4 Institute of Pathology, Erlangen University Hospital, Friedrich Alexander University of Erlangen–Nuremberg, Comprehensive Cancer Center Erlangen-EMN, Erlangen, Germany; 5 Institute of Human Genetics, Erlangen University Hospital, Friedrich Alexander University of Erlangen–Nuremberg, Comprehensive Cancer Center Erlangen-EMN, Erlangen, Germany; 6 Division of Hematology and Oncology, Department of Medicine, David Geffen School of Medicine, University of California at Los Angeles, Los Angeles, CA USA

**Keywords:** Endometriosis, Ovarian cancer, Endometrial cancer, Risk factor

## Abstract

**Background:**

No screening programs are available for ovarian or endometrial cancer. One reason for this is the low incidence of the conditions, resulting in low positive predictive values for tests, which are not very specific. One way of addressing this problem might be to use risk factors to define subpopulations with a higher incidence. The aim of this study was to investigate the extent to which a medical history of endometriosis can serve as a risk factor for ovarian or endometrial cancer.

**Methods:**

In a hospital-based case–control analysis, the cases represented patients with endometrial or ovarian cancer who were participating in studies aimed at assessing the risk for these diseases. The controls were women between the age of 40 and 85 who were invited to take part via a newspaper advertisement. A total of 289 cases and 1016 controls were included. Using logistic regression models, it was tested whether self-reported endometriosis is a predictor of case–control status in addition to age, body mass index (BMI), number of pregnancies and previous oral contraceptive (OC) use.

**Results:**

Endometriosis was reported in 2.1 % of the controls (*n* = 21) and 4.8 % of the cases (*n* = 14). Endometriosis was a relevant predictor for case–control status in addition to other predictive factors (OR 2.63; 95 % CI, 1.28 to 5.41).

**Conclusion:**

This case–control study found that self-reported endometriosis may be a risk factor for endometrial or ovarian cancer in women between 40 and 85 years. There have been very few studies addressing this issue, and incorporating it into a clinical prediction model would require a more precise characterization of the risk factor of endometriosis.

## Background

Ovarian cancer is associated with a high mortality rate in comparison with other cancers. In the United States, the incidence of ovarian cancer is estimated to be around 22,200 annually. About 14,000 of these women are expected to die of the disease [1]. In Germany the corresponding figures are 7400 and 5500 [2]. This high mortality rate is mainly the consequence of ineffective early detection or screening programs. Most of the cancers are diagnosed at advanced stages. Uterine endometrial cancer is the most frequent type of gynecological cancer. In Germany, there are approximately 11,600 new diagnoses every year and 2400 disease-related deaths [2]. Although the mortality due to endometrial cancer is fairly low, there are no established early detection methods or screening programs for this disease. Earlier detection would result in much less invasive surgery and less use of radiotherapy and chemotherapy, leading to substantial benefits for the patients.

With regard to ovarian cancer, effective risk-reducing strategies have been described. Bilateral salpingo-oophorectomy has been shown to reduce the risk among *BRCA* mutation carriers by 71–96 % [3–5]. Numbers of live births, oral contraceptive use, and tubal ligation are also associated with a significant reduction in the lifetime risk of ovarian cancer.

There are no established screening programs for endometrial cancer, but risk-modifying strategies are known that allow the risk of endometrial cancer to be controlled — such as weight control, physical activity, and no exogenous unopposed estrogen [6–9].

Risk factors are therefore of special interest for both diseases, since accurate risk prediction might make individualized early detection or screening programs possible. Risk factors for ovarian cancer include reproductive behavior and use of hormonal therapies. Pregnancies and the use of oral contraceptives can reduce the incidence of ovarian cancer [10]. Mutations in the *BRCA1* and *BRCA2* genes are reported to lead to a lifetime risk of about 20–40 and 15–25 %, respectively [11]. Large-scale genotyping efforts have recently identified and confirmed a total of 11 low-penetrance risk loci that are common in the population [12–20].

Endometrial cancer risk factors include hormonal and metabolic factors such as obesity, tamoxifen use, diabetes, hypertension, and high dietary fat consumption [21]. With regard to genetic risk factors, endometrial cancer is the most common malignancy in women, with mutations associated with Lynch syndrome [22]. Genome-wide association studies have identified some low-penetrance loci, but large-scale confirmation studies are still pending [23–25].

In this study endometriosis is evaluated as a risk factor for ovarian- or endometrial cancer. Endometriosis is a chronic disease that affects 4–30 % of all women during the reproductive age [26–28]. Furthermore it is one of the most frequent gynecological diseases. However it can reasonably be assumed, that the prevalence is about 10 % [28]. The pathogenesis of endometriosis is considered to be complex. Historically a metaplastic transformation of peritoneal cells or the still favourably retrograde menstruation of cells through the tubes into the peritoneal cavity are discussed [29]. On a molecular level different pathways such as the estrogen and progesterone pathway, vasculogenesis, sphingolipids, prostaglandins, and cytokines appear to be involved.

Pelvic pain during menstruation is the main symptom in patients with endometriosis. Other symptoms can be chronic lower abdominal pain, dysuria, dyschezia and/ or dyspareunia. The disease is characterized by endometrial cells outside the uterus and is located mainly in the retrouterine pouch. The diagnosis occurs in gynecological examination and especially during laparoscopic surgeries with histological verification [30]. Therapy options comprise mainly medication and surgical therapy. The surgical removal of the lesion is often the first line therapy [31].

An association between endometriosis and both diseases has been suggested, and in the case of ovarian cancer the connection is clearly established [32–35]. Patients with endometriosis tend to be younger and to be diagnosed at earlier stages and with lower-grade ovarian cancer lesions [36, 37]. With regard to endometrial cancer, the evidence is less clear. A reduced risk of endometrial cancer was even found in a nested case–control study including 39 patients with endometrial cancer and 211 controls (OR 0.58; 95 % CI, 0.42 to 0.81) [37]. In a different nested case–control study, patients were found to have a relative risk (RR) of 1.23 (95 % CI, 0.63 to 2.38) [38]. However, most of the relevant studies only include a small number of events, so that definitive conclusions about associations cannot as yet be drawn [39–42].

The aim of the present case–control study was to investigate the extent to which a medical history of endometriosis represents a risk factor for ovarian or endometrial cancer in addition to age, body mass index (BMI), number of pregnancies, and previous oral contraceptive (OC) use.

## Methods

A series of case–control and cohort studies have been conducted in the Department of Gynecology and Obstetrics at Erlangen University Hospital in an effort to identify risk factors for breast cancer and gynecological cancer, as well as prognostic factors. These are: 1. The Bavarian Ovarian Cancer Study (BAV) which was conducted from 2002 to 2011 and was affiliated to large-scale research consortia working on identifying genetic and epidemiologic risk factors [13–17, 19, 20], as well as prognostic factors [43–45]. 2. The Bavarian Endometrial Cancer Study (BECS) conducted from 2002 to 2013 also affiliated to larger research consortia [23–25]. 3. The Bavarian Breast Cancer Cases and Controls Study (BBCC) [17, 46–51] conducted from 2002 to 2013. The corresponding controls were recruited using local newspaper advertisements inviting women over the age of 40 without breast, ovarian or endometrial cancer anamnesis, respectively.

Cases of this study were patients with histologically confirmed current or former endometrial or invasive epithelial ovarian cancer disease who were treated at Erlangen University Hospital. The controls originate from the three studies mentioned above. Women who had any other types of cancer were not eligible for inclusion in the study. All subjects had to complete the same self-reported medical history form and the same study questionnaire. The age criteria of cases and controls were to be over 40 and less than 85 years. The ethics committee of the medical faculty at Friedrich Alexander University, Erlangen, approved the study and all of the patients and healthy participants provided written informed consent.

### Data acquisition

A standardized questionnaire including modules on pregnancy history, previous use of hormonal contraceptives and hormone replacement therapy, medical history, family history, and lifestyle was filled out by the patients and healthy control individuals, and was completed in a structured interview with trained medical personnel if any questions had not been fully answered. The question about a history of endometriosis was expressed in a “yes/no/don’t know” form, and was answered by cases and controls in the same way when completing the questionnaire. Additional information for patients was obtained from the patient charts, such as information about medical procedures, histology of the tumor, and concomitant medication.

### Statistical considerations

The primary objective was to investigate whether information about endometriosis can be used to assess the risk for ovarian or endometrial cancer, in addition to other well-known risk factors. For this purpose, a multiple logistic regression model was fitted with cancer case–control status as a binary outcome (yes vs. no) and the following predictors: endometriosis status (categorical; yes vs. no), age (continuous), BMI (continuous), number of pregnancies (integer), and oral contraceptive use (categorical; yes vs. no). The Wald test was performed for endometriosis status. A significant *P* value would indicate that endometriosis information is an additional risk factor for ovarian or endometrial cancer. The regression model was also used to estimate adjusted odds ratios (ORs), particularly for endometriosis status.

Patients for whom outcome data were lacking and patients with missing information on age or endometriosis were excluded. Missing predictor values were imputed using single “best guesses” (median value of continuous or integer predictors, the most common value of categorical or ordinal predictors) based on nonmissing data across all subjects. Continuous predictors were used as natural cubic spline functions to describe nonlinear effects [52]. The number of degrees of freedom (1 or 2) of each predictor was determined as done recently in [53].

The performance of the logistic regression model in terms of discrimination and calibration (“goodness of fit”) was assessed using the area under the receiver operating characteristic curve (AUC) and the Hosmer–Lemeshow statistic applied to the case–control design [54]. The AUC ranges from 0.5 (no discrimination between cases and controls) to 1 (perfect discrimination). It can be interpreted as representing the probability that the model will give a person who has disease a higher probability of being diseased than it gives to a randomly chosen healthy person. In accordance with Hosmer and Lemeshow, patients were ranked with respect to the predicted conditional probability of ovarian or endometrial cancer and categorized into equal-sized groups based on percentiles. Frequencies of predicted events in each group were compared with frequencies of observed events in each group using a scatter plot and the Hosmer–Lemeshow *χ*^2^ test. A large *P* value indicates satisfactory calibration.

Model building was evaluated by 10-fold cross-validation with 20 repetitions to address overfitting. For this purpose, the model-building process (i.e., determination of cubic spline functions and estimation of regression coefficients) was carried out on each training set, resulting in several logistic regression models (one model per set), which were then used to calculate the AUCs on the corresponding validation data sets. The average of all these AUCs was taken as an evaluation measure. This cross-validated AUC may be regarded as an estimation of the expected probability of two randomly chosen future ill or healthy subjects being correctly classified as ill or healthy, respectively, using the main regression model described above.

As sensitivity analysis, a simple logistic regression model was fitted to get an unadjusted OR for endometrioses status.

All of the tests were two-sided, and a *P* value of < 0.05 was regarded as statistically significant. Calculations were carried out using the R system for statistical computing (version 3.0.1; R Development Core Team, Vienna, Austria, 2013).

## Results

### Descriptive statistics

A total of 1305 participants were included in the analyses, of whom 165 were patients with ovarian cancer, 131 were patients with endometrial cancer, and 1016 were control individuals. Complete information with all variables was available for 90 % of the participants. The proportions of missing predictor values were between 5.5 and 6.5 %. The missing values were imputed, as described above. Descriptive statistics are shown in Table 1. Endometriosis was noted by 2.1 % of the controls (*n* = 21) and by 4.8 % of the cases (*n* = 14). The mean age of subjects with endometriosis was 53.2 years for cases and 57.7 years for controls. Endometriosis was present in 4.2 % of the ovarian cancer patients (seven of 165 patients) and in 5.3 % of the endometrial cancer patients (seven of 131 patients).

**Table 1:** Characteristics of the study participants, showing mean and standard deviation (SD) for continuous characteristics and frequency and percentage for categorical characteristics

Characteristic	Controls (*n* = 1016)		Cases (*n* = 289)		Ovarian cancer cases (*n* = 165)		Endometrial cancer cases (*n* = 131)^a^	
	Mean or n	SD or %	Mean or n	SD or %	Mean or n	SD or %	Mean or n	SD or %
Age [years]	60.9	9.3	62.1	11.1	59.5	11.1	65.6	10.5
Body mass index [kg/m^2^]	25.5	4.3	27	5.8	26	4.7	28.3	6.9
Self-reported endometriosis								
No	995	97.9	275	95.2	158	95.8	124	94.7
Yes	21	2.1	14	4.8	7	4.2	7	5.3
Oral contraceptive use								
No	275	27.1	141	48.8	72	43.6	71	54.2
Yes	741	72.9	148	51.2	93	56.4	60	45.8
Pregnancies (n)								
0	121	11.9	41	14.2	14	8.5	27	20.6
1	165	16.2	62	21.5	31	18.8	32	24.4
2	373	36.7	100	34.6	66	40.0	37	28.2
3	219	21.6	52	18.0	35	21.2	19	14.5
4+	138	13.6	34	11.8	19	11.5	16	12.2

^a^Summed up numbers of ovarian and endometrial cancer cases is larger than 289, because there were cases with both ovarian and endometrial cancer

### Prediction of ovarian or endometrial cancer

The preliminary logistic regression analyses showed that the continuous predictors of age and BMI fitted best as cubic spline functions both with two degrees of freedom. The main logistic regression analyses indicated that endometriosis status is a risk factor for ovarian or endometrial cancer (*P* < 0.01, Wald test), in addition to well-known risk factors. Women with a history of endometriosis had an increased risk of developing ovarian or endometrial cancer when all other predictors were also considered (Table 2).

**Table 2:** Logistic regression analyses, showing adjusted^a^ odds ratios (ORs), with the corresponding 95 % confidence intervals (CIs) in brackets

Predictor		OR (95 % CI)
Age^b^	Younger vs. medium	1.36 (1.11, 1.66)
	Older vs. medium	1.24 (1.07, 1.43)
	Older vs. younger	0.91 (0.70, 1.18)
BMI^c^	Low vs. medium	0.99 (0.83, 1.17)
	High vs. medium	1.26 (1.09, 1.46)
	High vs. low	1.28 (0.95, 1.72)
Oral contraceptive use	Yes vs. no	0.43 (0.32, 0.58)
No. of pregnancies	Per-pregnancy increase	0.93 (0.84, 1.02)
Self-reported endometriosis	yes vs. no	2.63 (1.28, 5.41)

*BMI* body mass index

^a^ORs were estimated using a multiple logistic regression model, with the predictors listed in the first column of the table

^b^Age was used as a nonlinear continuous predictor. It was evaluated at the first sextile (“young” — i.e., 51 years), median (“medium” — i.e., 62 years), and fifth sextile (“older” — i.e., 70 years)

^c^BMI was used as a nonlinear continuous predictor. It was evaluated at the first sextile (“low” — i.e., 21.7 kg/m^2^), median (“medium” — i.e., 25.0 kg/m^2^), and fifth sextile (“high” — i.e., 30.1 kg/m^2^)

Oral contraceptive use was protective, but the number of pregnancies did not appear to influence the risk of cancer in this study. Both younger women and older women had a higher risk than medium-aged women. There were no relevant differences between older and younger women. Women with a high BMI had a higher risk than women with a medium or low BMI. There were no relevant differences between women with a low and medium BMI (Table 2).

**Fig. 1:**
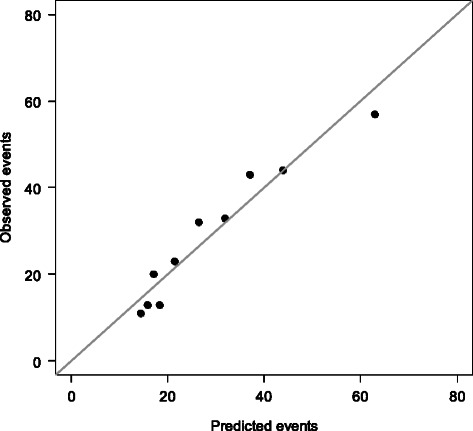
Observed and predicted frequencies of ovarian or endometrial cancer cases. The patients were ranked according to the predicted conditional probability of being a case by the logistic regression model, and grouped into 10 categories based on deciles. Numbers of observed cancer cases in each category (“observed events”) are plotted against the summed-up predicted probabilities of being a case in each category (“predicted events”). Points below the gray line indicate when the regression model overestimates the cancer risk, and points above it indicate underestimation. A perfect prediction model would have all points on the gray line

The logistic regression model appeared to be well-calibrated (*P* = 0.44, Hosmer–Lemeshow *χ*^2^ test). The AUC on the whole data set was 0.685; the cross-validated AUC was slightly smaller (0.675), indicating slight overfitting. Figure 1 shows that there was a good correlation between the observed frequencies of ovarian or endometrial cancer cases and the frequencies predicted by the regression model.

The sensitivity analysis yielded a similar result. The unadjusted OR for endometriosis status was 2.41 (95 % CI, 1.21 to 4.81) indicating that the predictors of the multiple regression model behaved unsuspiciously.

## Discussion

In this case–control study, self-reported endometriosis was confirmed as a risk factor for a combined group of ovarian or endometrial cancer patients between the age of 40 and 85. In addition, other already well-known risk factors for ovarian and endometrial cancer such as age and BMI were confirmed.

Endometriosis has been identified as a risk factor for subtypes of ovarian cancer [55, 56]. In a large, multicenter study including more than 1500 patients with endometriosis, 7900 patients with ovarian carcinoma and 13,200 control patients, endometriosis was identified as a risk factor for clear cell, endometrioid, and low-grade serous ovarian carcinoma [57]. Clear increases in risk were found in the group of endometriosis patients for clear cell ovarian carcinoma (OR 3.75; 95 % CI, 3.04 to 4.58), for endometrioid ovarian carcinoma (OR 2.32; 95 % CI, 1.94 to 2.78), and for low-grade serous ovarian carcinoma (OR 2.02; 95 % CI, 1.38 to 2.97). These findings did not apply to high-grade serous ovarian carcinoma (OR 1.11; 95 % CI, 0.96 to 1.29). In a national in-patient registry in Sweden from 1969 to 1983 a cohort of 64,492 patients with a hospital diagnosis of endometriosis was also found to have a significantly elevated risk for ovarian cancer [58].

Until now, endometriosis has not been defined as a risk factor for endometrial cancer. There are currently no data from population-based studies suggesting an association between endometriosis and endometrial cancer. A retrospective case–control study including 1399 patients did not show any association between endometrial cancer and endometriosis [59]. Previously reported data on endometriosis as a risk factor for endometrial cancer are inconclusive [37–42]. The studies mentioned have limited case numbers in comparison with the present study, which confirmed an increased risk.

As mentioned above, an increased risk of epithelial ovarian cancer in patients with endometriosis has been shown in numerous epidemiologic studies, but the pathogenesis is poorly understood [35]. Current molecular studies have sought to link the two conditions via pathways related to oxidative stress, inflammation, and hyperestrogenism. As a result of repetitive hemorrhage, with an accumulation of heme and free iron in endometriotic lesions, reactive oxygen species are produced and play a role in the development of ovarian carcinoma [60]. Similarly, cytokines and mediators are responsible for the microenvironment of endometriosis and endometriosis-associated ovarian carcinoma.

Although endometriosis is not yet established as a risk factor for endometrial cancer, recent studies have discussed an influence of the epithelial-to-mesenchymal transition and stem cells in endometrial cancer [61]. Endometrial stem cells are frequent in endometrial tissue during menstruation. It may therefore be speculated that endometrial stem cells may play an important role in the development of endometriotic implants [62] and thus in endometriosis and endometrial cancer.

A molecular pathway cable of confirming the hypothesis is not currently known. An epigenetic analysis has identified *HNF1B* as a subtype-specific susceptibility gene for ovarian cancer [16]. Different variants in *HNF1B* are associated with the risk of serous or clear cell epithelial cancer. *HNF1B* is also overexpressed in endometriosis [16], supporting the hypothesis that the gene may have an oncogenic role in initiating specific subtypes of ovarian cancer in patients with endometriosis. *HNF1B* might also be the link to endometrial cancer. A genome-wide association study has linked minor alleles of certain single nucleotide polymorphisms in *HNF1B* with a decreased risk of endometrial cancer [23]. Further research is needed in order to define a molecular pathway.

It has been hypothesized that endometriosis develops from stem/progenitor cells. It would be of great interest to associate the technique for identifying stem/progenitor cells in endometriotic tissues with an analysis of genetic/epigenetic changes in these cells that may possibly affect their molecular signature and activity [63]. This might make it possible to identify a molecular pathway for the development of ovarian or endometrial cancer in patients with endometriosis.

This study is the first case–control study to confirm the influence of endometriosis on ovarian and endometrial cancer in a population in Germany. One advantage of this case–control study was the validated epidemiological questionnaire that was used. A limitation is the small number of cases, due to the low incidences of ovarian and endometrial cancer, at 18.6 patients per 100,000 population and 26.9 patients per 100,000 population, respectively. Similarly in this study there are fewer patients with reported endometriosis than expected by a prevalence of 10 % in reproductive age. This effect can be caused by a notoriously underdiagnosed disease and the prespecified age range from 40 to 85 in our cases and controls with a consecutively decrease in symptoms [64], which leads probably to a reduced description in the medical history form and the study questionnaire. The number of 2.1 % endometriosis in controls respectively 4.8 % in cases is congruent with the data of the Iowa Women’s Health Study with a cohort of more than 40,000 postmenopausal women, which publicated a number of 3.8 % of self-reported history of endometriosis [40]. Self-reported endometriosis is an inexact and inaccurate method of assessment and may force up the case numbers for endometriosis. Also the higher number of self-reported endometriosis in patients with ovarian- or endometrial-cancer could originate in a better knowledge and remembering of their previous gynecological diseases. Further limitations are the retrospective analysis of the data and the combined analysis of ovarian and endometrial cancer cases. The reason for the combined analysis was the low rate of seven patients with endometriosis in each group of patients with ovarian (*n* = 158) or endometrial cancer (*n* = 124). Statistical analyses were performed for each group, and there was a significantly higher risk in the group of patients with endometrial cancer and no significance in the patients with ovarian cancer. However, these data are not shown, due to the small number of cases of endometriosis in each group. Our results do not necessarily hold for subjects younger than 40, because women of this age were excluded from this study.

## Conclusions

There have been few studies addressing the question of whether endometriosis is a risk factor for ovarian or endometrial cancer, and incorporating this into a clinical prediction model would require precise characterization of endometriosis as a risk factor. Larger studies are needed in order to confirm the data for subgroups - especially for a younger population than the described one -, to examine molecular pathways, and to understand the pathogenesis.

## Abbreviations

AUC: Area under the receiver operating characteristic curve
BAV: Bavarian Ovarian Cancer Study
BBCC: Bavarian Breast Cancer Cases and Controls Study
BECS: Bavarian Endometrial Cancer Study
BMI: Body mass index
OC: Oral contraceptive
RR: Relative risk
OR: Odds ratio
